# The role of interleukins in peripheral nerve injury and the current status of treatment

**DOI:** 10.3389/fimmu.2025.1691335

**Published:** 2026-01-07

**Authors:** Yue Shen, MingZhu Li, ShengBo Jin, ZiYang Yu, Qian Liu, HuiNi Yao, YuXin Jiang, JiaQing Fu, NingXin Li

**Affiliations:** 1 Integrated Traditional Chinese and Western Medicine Department, Cancer Hospital of China Medical University, Shenyang, Liaoning, China; 2 Integrated Traditional Chinese and Western Medicine Department, Liaoning Cancer Hospital & Institute, Shenyang, Liaoning, China; 3 Acupuncture and Massage Department, Cancer Hospital of Dalian University of Technology, Shenyang, Liaoning, China; 4 College of Acupuncture and Massage, Liaoning University of Traditional Chinese Medicine, Shenyang, Liaoning, China

**Keywords:** interleukin, peripheral nerve injury, cell therapy, physical therapy, gene therapy

## Abstract

Peripheral Nerve Injury (PNI) is a common condition in modern clinical practice, characterized by a high incidence and significant debilitating consequences. This narrative review systematically delineates the dual functions of key pro-inflammatory (e.g., IL-1β, IL-6, IL-17) and anti-inflammatory (e.g., IL-4, IL-10, IL-13) interleukins in the spatiotemporal context of nerve injury. This is a narrative review, the review systematically summarizes the dual pro-inflammatory and anti-inflammatory roles of interleukins in peripheral nerve injury. The therapeutic potential of interleukin-based treatment strategies, including inhibitors, agonists, and their combined applications with cell therapy, gene therapy, physical therapy, etc., was explored, and the limitations of current research and future research directions were pointed out. By synthesizing current evidence, this review aims to provide a comprehensive mechanistic overview and highlight emerging interleukin-centric therapeutic paradigms for PNI.

## 1 Introduction

Peripheral nerve injury (PNI) has emerged as a significant clinical issue due to its high incidence and considerable disabling consequences. The pathogenesis of PNI is multifactorial, encompassing both traumatic factors, such as compression, transection, and traction (1), as well as non-traumatic factors, including metabolic disorders, neurotoxic substances, and disturbances in nerve conduction (2). Patients commonly present with sensory abnormalities in the affected limbs, such as numbness, tingling, burning sensations, or intense pain (3). In severe cases, PNI can lead to the loss of both motor and sensory functions, resulting in physical disability and intractable neuropathic pain, thereby severely affecting the patient’s quality of life (4). Despite the advances in modern surgical repair techniques, including direct nerve repair, autologous nerve grafting, and cadaveric allografting, approximately one-third of PNI cases exhibit incomplete recovery and suboptimal functional restoration (5). Various non-surgical approaches, such as pharmacological treatments, electrical stimulation, cellular therapies, and laser therapies, have been explored to promote myelination and enhance functional recovery following peripheral nerve injury (6); however, their effects remain limited. Although PNI has been extensively studied, effective clinical treatment options remain scarce (7).

Immunological cells like lymphocytes and macrophages are the main producers of interleukins (ILs), which are essential for controlling inflammation, tissue repair, and immunological homeostasis (8). There are currently about 40 different kinds of interleukins known to exist (9). These can be classified into pro-inflammatory ILs (e.g., IL-1β, IL-6, IL-17) and anti-inflammatory/pro-repair ILs (e.g., IL-4, IL-10, IL-13), based on their predominant functions. In the spatiotemporal dynamics following peripheral nerve injury (PNI), the expression and balance of different interleukins directly influence the outcome of nerve repair.

Among the myriad molecules that coordinate immune responses following nerve injury, the interleukin family occupies a central and pivotal role (10). The immune regulatory mechanisms centered around interleukins have a profound impact on nerve repair processes. For instance, T-helper (Th) cells precisely regulate macrophage function by secreting specific interleukins, enabling macrophages to acquire anti-inflammatory and pro-regenerative properties. This significantly enhances the microenvironment for nerve regeneration (11). It is worth noting that the regulatory role of interleukins is not simply “good or bad”. Its functions exhibit a high degree of spatiotemporal dynamics and situational dependence. In the early stages of injury, innate immune cells, such as neutrophils, macrophages, and lymphocytes, rapidly infiltrate the site of injury and release relevant interleukins, thus contributing to the repair process (12).

This study reviews and synthesizes the regulatory role of interleukins in peripheral nerve injury, focusing on the timing and mechanisms of action of both pro-inflammatory and anti-inflammatory interleukins. We will examine the regulatory mechanisms of pro-inflammatory and anti-inflammatory interleukins, emphasizing their time-dependent and context-specific roles in nerve degeneration, pain, and regeneration. We will then synthesize the current landscape of interleukin-targeted therapeutic strategies, including inhibitors, agonists, and their innovative combinations with other modalities such as neurotrophic factors, cell therapy, and physical stimulation. The overarching aim is to elucidate the critical role of interleukins in PNI and evaluate their potential as therapeutic targets for improving peripheral nerve repair.

## 2 Research strategy

### 2.1 Search strategy

We conducted a comprehensive literature search in the following electronic databases from 2005 to 2025, with the search time limit up to October 2025,including systematic queries of electronic databases such as PubMed and the China National Knowledge Infrastructure (CNKI). The search strategy was designed to include a combination of keywords and Medical Subject Headings (MeSH) terms related to “interleukin,” “peripheral nerve injury,” “nerve regeneration,” “neuroinflammation,” and specific interleukins (e.g., IL-1β, IL-6, IL-10, IL-4, IL-13, IL-17).

### 2.2 Eligibility criteria

Select research based on the following PICOS framework:

Population (P): Individuals affected by peripheral nerve injury (PNI), including patients with traumatic (such as compression, rupture) and non-traumatic (such as metabolic disorders, exposure to neurotoxic substances) causes.

Characteristics: It may involve different disease course stages (acute/chronic), age, gender and comorbidities, but no specific subgroups are defined.

Intervention (I):

Treatment regimens targeting ILs: Inhibitors: such as IL-1β inhibitors (Anakinra), IL-6 inhibitors (Tocilizumab), and IL-17A inhibitors (Secukinumab).

Agonists: such as IL-4/IL-13 agonists, IL-10 agonists.

Combined application of ILs with other therapies:Neurotrophic factor (NGF/BDNF), stem cell therapy, electrical stimulation, and gene therapy (such as AAV delivery of IL-10 and GDNF).

Comparison (C):

Reference Settings:

Conventional treatment: surgical repair (autologous nerve transplantation), traditional drugs (such as antiepileptic drugs).

Placebo or no intervention: Observe the natural course of the disease or only use supportive treatment (such as analgesics).

Outcome (O)

Main outcome:Neurological function recovery: Motor/sensory function scores (such as the MNSI scale), improvement in nerve conduction velocity.

Pain relief: Results of Visual Analogue Scale (VAS) and mechanical hyperalgesia test.

Inflammatory markers: Changes in serum/tissue concentrations such as IL-1β, IL-6, TNF-α, etc.

Histological improvement: Reduced Schwann cell activity, myelin formation and glial scarring.

Secondary outcomes: Quality of life score, incidence of complications, and treatment tolerance.

Study Design (D)

Research type:

Clinical research: Randomized controlled trials (RCTs), case-control studies, prospective cohort studies.

Basic research: Animal model experiments (such as rat sciatic nerve injury models), *in vitro* cell experiments.

Systematic review: A meta-analysis or narrative summary of existing evidence.

Exclusion criteria include: conference abstracts, editorials, case reports, studies that do not focus on peripheral nerves, and studies where full texts cannot be obtained.

### 2.3 Study selection

All retrieved records were imported into EndNote X9 for duplicate removal. The study selection process was performed independently by two reviewers (Y.S. and M.L.) in two stages:

1. Title and Abstract Screening: Potentially relevant articles were identified based on the eligibility criteria.

2. Full-Text Review: The full texts of the selected articles were assessed for final inclusion.

Any disagreements between the reviewers were resolved through discussion or by consulting a third reviewer (S.J.).

### 2.4 Data extraction

Data from the included studies were extracted independently by two reviewers using a pre-piloted data extraction form in Microsoft Excel. The extracted data included: Study characteristics (author, year, country)、Animal/cell model used, Type of nerve injury、Interleukins investigated、Key findings related to expression timing, mechanisms, and functional outcomes (pro-inflammatory vs. anti-inflammatory effects)、Main conclusions.

**Figure 1:**
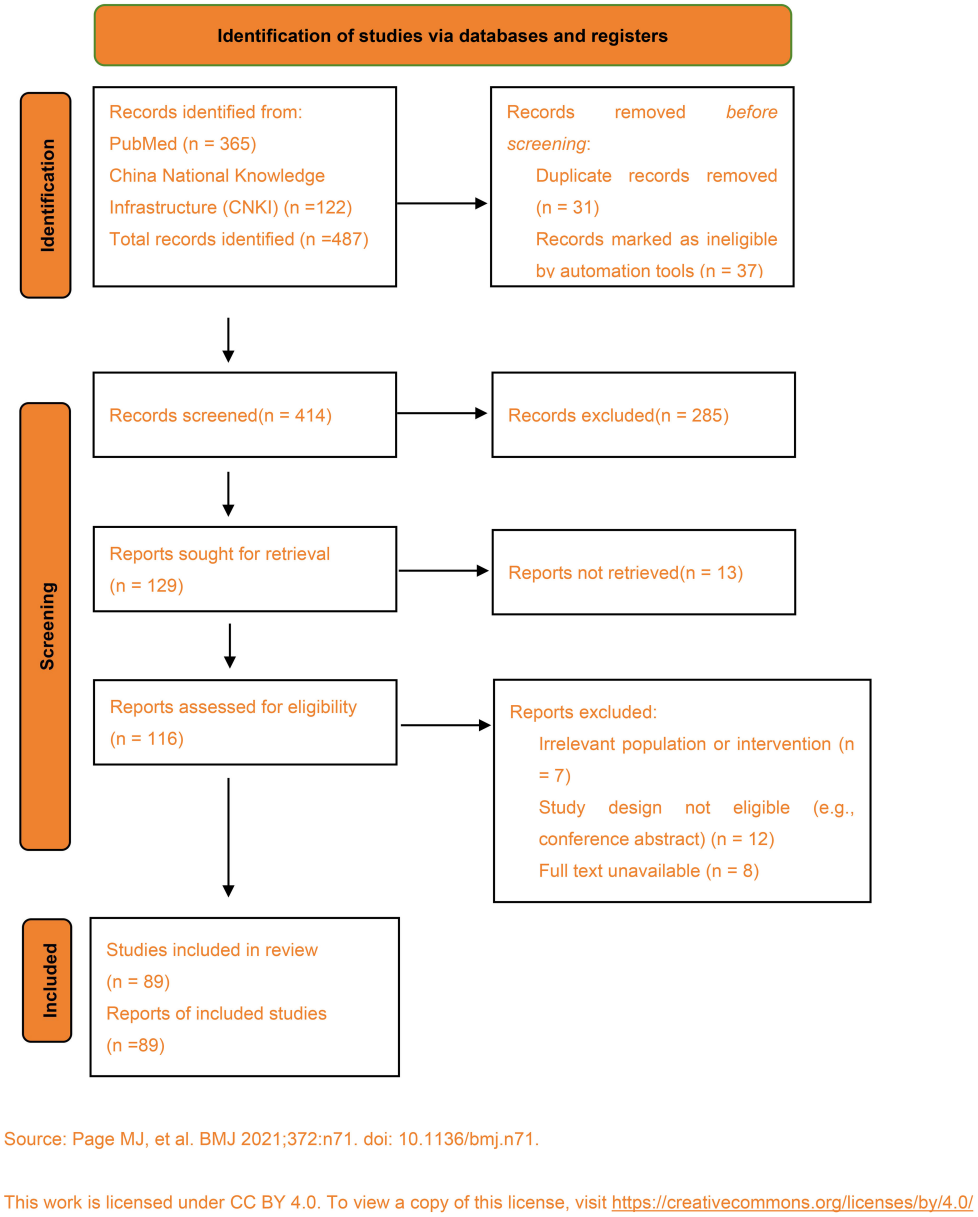
Indentification of studies via databases and registers. Source: Page MJ, et al. BMJ 2021; 372:n71. doi:10.1136/bmj.n71.

A detailed summary of the research selection process is available. The PRISMA flowchart is shown in **Figure 1**.

## 3 The regulatory role of interleukins in peripheral nerve injury

### 3.1 The role of pro-inflammatory interleukins

Pro-inflammatory interleukins, such as IL-1β, IL-6, and IL-17, play a pivotal role in the pathophysiology of peripheral nerve injury. The regulatory effect of interleukins is highly spatiotemporal dynamic and context-dependent, After peripheral nerve injury, Wallerian Degeneration (WD) is an early key process, involving the degradation of axons and myelin sheaths, inflammatory cell infiltration, and phenotypic transformation of Schwann cells (SCs). Pro-inflammatory interleukins are released by activated Schwann cells and infiltrating macrophages (13). These interleukins contribute to local inflammatory responses by activating distinct signaling pathways., In the early stage, it can initiate repair by removing necrotic tissue, while in the chronic stage, This cascade of inflammatory events not only exacerbates secondary injury following nerve damage but also impedes nerve repair and regeneration mechanisms (14).

#### 3.1.1 Interleukin-1 β

IL-1β is primarily produced by peripheral immune cells and blood-derived leukocytes. However, it can also be synthesized and released by microglia, astrocytes, and even neurons within the brain and spinal cord (15). Studies have demonstrated that the level of IL-1β remains low within the first hour following nerve injury. It increases two-fold by 6 hours, peaks ten-fold at 24 hours, and then gradually declines, maintaining elevated levels for several days before returning to baseline around 14 days (16).

##### 3.1.1.1 Expand the inflammatory response

Schwann cells release chemokines like CCL2 and CXCL1 following nerve damage, which starts an inflammatory chain reaction. For example, macrophages are drawn to the site of injury when CCL2 is upregulated (17). By releasing pro-inflammatory cytokines like IL-1β, these invading macrophages then intensify the inflammatory response even more. CXCL1, on the other hand, binds to CXCR2 on the surface of macrophages, promoting their migration to the affected area. These macrophages activate inflammatory pathways, including the NLRP3 inflammasome (18), leading to the processing of pro-inflammatory cytokines, such as IL-1β, into their active forms and their subsequent secretion into the extracellular space (19). This exacerbates the local inflammatory response. The sustained and amplified inflammation not only accelerates macrophage recruitment and enhances the infiltration of neutrophils and monocytes but also exerts toxic effects on neuronal cells, thereby impeding peripheral nerve regeneration (20).

##### 3.1.1.2 Inhibit nerve regeneration

Schwann cells are the myelin-forming cells of the peripheral nervous system, and their functional integrity is critical for maintaining normal neuronal function. When the structure of Schwann cells or their myelin sheaths is disrupted, or when their function is impaired, it can lead to peripheral neuropathy (21). Notably, The rapid increase of IL-1 within hours to days after injury is an early event necessary to initiate Wallerian degeneration and recruit macrophages to clear axon and myelin debris (22).after injury, Schwann cells undergo dedifferentiation, transforming from a mature myelin maintenance state to an immature repair phenotype (“dedifferentiated SCs”). This process is triggered by axon degradation, causing Schwann cells to dissociate from axons and initiate migration to clear debris (i.e., Wallerian degeneration). Dedifferentiated Schwann cells are called “repair Schwann cells”, and their core feature is to recruit macrophages by secreting cytokines and chemokines, thereby promoting the formation of neural Bridges and axon regeneration (23). The contradiction lies in that if this process gets out of control, the continuous high expression of IL-1β will transform from a “scavenger” to a “destroyer”.Pyroptosis is the main mechanism of Schwann cell death in this diseased setting. Peripheral nerve injury triggers Schwann cell pyroptosis, which in turn releases inflammatory mediators, such as IL-1β. Pyroptotic Schwann cells directly impair neuronal function, while inflammatory mediators further propagate pyroptosis in surrounding cells by amplifying the inflammatory response, thus creating a vicious cycle. Persistent inflammation and pyroptosis hinder nerve repair, resulting in delayed peripheral nerve regeneration (24). Moreover, IL-1β can regulate astrocyte proliferation by activating the JAK-STAT signaling pathway, promoting the growth of new fibers, exacerbating glial scar formation, and impeding axonal extension (25), Ultimately, a vicious cycle of “inflammation - injury - aplastic disorder” is formed.

##### 3.1.1.3 Intensify the development of pain

Peripheral nerve injury (PNI) drives the onset and progression of neuropathic pain through a dual mechanism. First, the injury activates Toll-like receptors, triggering the NF-κB signaling pathway, which directly regulates the expression of IL-1β in the dorsal root ganglion (DRG) and dorsal horn (26). Second, injury upregulates the TRPV1 receptor channels in nociceptors (27), further amplifying pain signals. IL-1β, a microglia-derived mediator, promotes the activation of spinal microglia, enhancing excitatory synaptic transmission in the superficial dorsal horn while simultaneously weakening inhibitory synaptic transmission. This imbalance leads to the transmission of nociceptive information and exacerbates the erroneous processing of sensory signals, resulting in central sensitization at both the spinal and supraspinal levels (28). Ultimately, this contributes to the persistent development of neuropathic and inflammatory pain.

#### 3.1.2 Interleukin-6

IL-6 is a multifunctional, pleiotropic cytokine that plays a key role in the regulation of immune responses, acute-phase responses, and inflammatory processes (29). In the early stages of injury (within 3 hours), Schwann cells are the primary source of IL-6, which is crucial for the chemotaxis of immune cells during the first 2–5 hours post-injury. In the later stages (2–5 days), macrophages and fibroblasts become the predominant sources of IL-6 (30), with levels peaking and remaining elevated until day 21 (31).

##### 3.1.2.1 Intensify the inflammatory response

At early time points following nerve injury, interleukin-6 (IL-6) plays a crucial role in driving the inflammatory cascade, exacerbating nerve damage. As a key mediator, IL-6 rapidly recruits macrophages to the site of injury (30), promoting their polarization towards the classically activated M1 phenotype (32). Additionally, IL-6 can stimulate phagocytes and monocytes to release more IL-6 into the bloodstream, thereby amplifying the local inflammatory response through chemokines while also triggering systemic immune activation (33). Furthermore, IL-6 can activate the signal transducer and activator of transcription 3 (STAT3) pathway, which leads to the upregulation of various inflammatory mediators (34), further intensifying the inflammatory response. Persistent inflammatory conditions can alter the cellular microenvironment, impairing nerve recovery and aggravating secondary nerve injury.

##### 3.1.2.2 Enhanced pain signals

When nerves are injured, dorsal root ganglion (DRG) neurons and satellite glial cells synthesize and release interleukin-6 (IL-6), thereby initiating inflammatory pathways through the activation of the JAK-STAT signaling cascade. On one hand, the persistent activation of the JAK-STAT pathway leads to the hyperexcitability of injured neurons, which contributes to the development of mechanical allodynia and thermal hyperalgesia. On the other hand, IL-6 facilitates the bilateral and systemic propagation of inflammatory signals, enabling the widespread transmission of neuroinflammatory responses and promoting the onset of pain (35).

##### 3.1.2.3 Hindering nerve regeneration

In peripheral nerve injury, the pro-inflammatory cytokine IL-6 plays a critical role in modulating nerve repair and regeneration through various mechanisms. First, IL-6 directly disrupts Schwann cell function by enhancing oxidative stress, compromising axonal integrity, and initiating a vicious cycle of neuroinflammation and neurodegeneration (36). Second, in the context of chronic nerve injury, persistently elevated IL-6 induces fibroblasts to undergo epithelial-mesenchymal transition (EMT) and fibroblast-myofibroblast transition (FMT). The resulting excessive fibrosis creates a mechanical barrier that obstructs nerve axon regeneration and impairs myelin sheath repair (37).

##### 3.1.2.4 Bidirectional regulatory effect

At early time points following nerve injury, denervated Schwann cells recruit macrophages to the injured site by releasing inflammatory factors such as IL-6. These factors act in concert with nerve membrane cells (denervated Schwann cells, or DSCs) to facilitate the phagocytosis of degenerated axonal and myelin debris (38). In this context, a moderate inflammatory response is beneficial for tissue clearance. However, excessive inflammation can lead to the sustained activation of immune-inflammatory cells and the subsequent degeneration of nerve cells (39). During the chronic phase of nerve injury, persistently elevated levels of IL-6 cooperate with macrophages to activate the STAT3 and STAT6 signaling pathways. This activation promotes the secretion of substances, such as lysosomal enzymes (e.g., CTSB), that degrade axonal growth inhibitory factors within the extracellular matrix (ECM), thus inhibiting axonal regeneration (40). This transition from “early promotion of repair to late inhibition of regeneration” highlights the bidirectional regulatory effects of IL-6-mediated macrophage function, shifting from protective clearance to destructive degradation.

#### 3.1.3 Interleukin

IL-17 is primarily produced by T helper 17 (Th17) cells, as well as neutrophils, mast cells, and natural killer (NK) cells (41). It plays a crucial role in various processes, including immune defense, tissue repair, and the pathogenesis of inflammatory diseases. Studies have demonstrated that IL-17 levels significantly increase 3 to 7 days following nerve injury, with elevated levels persisting throughout the chronic phase (42).

##### 3.1.3.1 Recruitment of immune cells

Interleukin-17 (IL-17) plays a pivotal role in the pathogenesis of various inflammatory diseases by facilitating the recruitment of immune cells to sites of injury. Specifically, the IL-17B/IL-17 receptor B (IL-17RB) signaling axis is critical for regulating chemokine production in Schwann cells. Activation of the IL-17B/IL-17RB pathway in Schwann cells induces the upregulation of multiple factors that are instrumental in macrophage recruitment, including CCL2, CCL3, and CCL5 (14). Moreover, IL-17 enhances the expression of chemokines and adhesion molecules in endothelial cells, promotes neutrophil infiltration, exacerbates neuroinflammation, and further disrupts the integrity of the blood-brain barrier.

##### 3.1.3.2 Inhibit nerve regeneration

Similar to IL-1β and IL-6, the function of IL-17 is also context-dependent. Interleukin-17 (IL-17) plays a key role in the initial injury clearance by recruiting neutrophils and macrophages and mediating myelin clearance and axon regeneration in the early stage of peripheral nerve injury. However, in the chronic stage, its pathological effect is also significant (14). IL-17 induces Schwann cells to secrete the pro-inflammatory protease MMP-9 via the STAT3 signaling pathway. MMP-9 can degrade the extracellular matrix, promoting the infiltration of inflammatory cells and exacerbating nerve injury. This process amplifies the inflammatory response in peripheral nerves and indirectly impairs the myelin regeneration function of Schwann cells (43). Additionally, IL-17 binds to IL-17 receptors (IL-17RA/RC) on the surface of fibroblasts, triggering downstream signaling pathways such as JAK-STAT, MAPK, and NF-κB. These pathways induce fibroblast proliferation, migration, and extracellular matrix (ECM) synthesis, resulting in endoneurial fibrosis, which hinders nerve regeneration (44). Furthermore, IL-17 exerts direct effects on Schwann cells (SCs). As a critical component in peripheral nerve myelination and remyelination following injury, IL-17 can directly promote demyelination mediated by the spinal cord cortex. By reprogramming stem cell differentiation, IL-17 diminishes their myelination capacity while enhancing their inflammatory functions, leading to inflammatory disorganization of the spinal cord cortex (45), thereby impairing both myelin regeneration and the functional recovery of peripheral nerves.

##### 3.1.3.3 Increased pain

The upregulation of IL-17 plays a pivotal role in the development of pathological pain by facilitating neuroinflammation, enhancing the excitability of dorsal root ganglion (DRG) neurons, and promoting communication between glial cells and neurons in the spinal cord. During the progression of pathological pain, IL-17 expression is significantly elevated in the spinal cord, activating spinal astrocytes via transient receptor potential (TRP) channels and kinin B1 receptors (B1R), as well as microglia, while also directly interacting with neurons. The activation of pro-inflammatory pathways through microglial IL-17 receptors (IL-17R) exacerbates pain (41). Moreover, IL-17 induces the release of pro-nociceptive mediators, including tumor necrosis factor-alpha (TNF-α) and interleukin-1 beta (IL-1β), as well as chemokines such as CXCR1/2 ligands, by activating key signaling pathways. This triggers an inflammatory cascade that alters neuronal sensitivity and mediates inflammatory pain (43).

**Figure 2:**
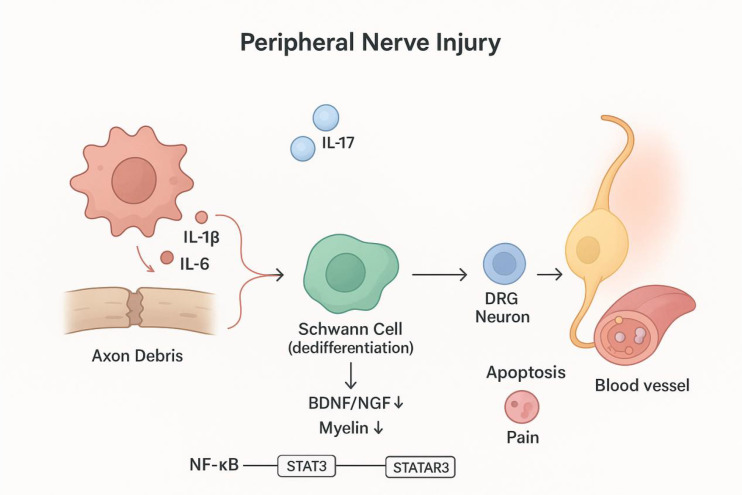
It can be seen from the figure that nerve injury triggers an inflammatory response, releasing pro-inflammatory cytokines such as IL-17 and IL-6. They may drive the dedifferentiation of Schwann cells by activating signaling pathways such as NF-kB and STAT3. Dedifferentiated Schwann cells down-regulate the expression of neurotrophic factors (such as BDNF and NGF), reduce the formation of myelin, and are not conducive to the repair and regeneration of axons. The diagram was drawn by the authors using Bio Render.

Considering the significant role of these pro-inflammatory interleukins (IL-1β, IL-6, IL-17) in exacerbating nerve injury, inflammation and pain, targeting their pathways provides therapeutic strategies for reducing secondary injury after PNI and improving prognosis. To more clearly explain the source, expression time series and mechanism of action of pro-inflammatory interleukins, the summary is shown in **Tables 1**–**3**. The mechanism of action is shown in **Figure 2**.

**Table 1:** The role of IL-1β.

Cytokine	Main source	Express the sequence of time	Mechanism of action		Quoted
IL-1β	Immune cells, microglia, astrocytes, neurons	The level of IL-1β is low 1 hour after nerve injury, increases two-fold by 6 hours, reaches its peak (ten-fold) at 24 hours, gradually declines after maintaining high levels for several days, and returns to baseline around 14 days	Expand the inflammatory response	Induce Schwann cells to secrete chemokines, amplifying local inflammatory responses.	(17)
				Activate the NLRP3 inflammasome, releasing mature IL-1β, and recruiting neutrophils and monocytes for infiltration, thereby hindering peripheral nerve regeneration.	(18–20)
			Inhibit nerve regeneration	Directly inhibit Schwann cell proliferation and myelination regeneration.	(24)
				Induce pyroptosis in Schwann cells.	(24)
				Promote astrocyte proliferation, exacerbating scar formation.	(25)
			Intensify the development of pain	Activate neurons in the dorsal root ganglion (DRG), triggering neuropathic pain.	(26, 27)
				Promote the activation of spinal microglia, enhancing central sensitization.	(28)

Concise synthesis of IL-1β’s regulatory mechanisms in peripheral nerve injury (PNI), specifically highlighting cytokine engagement, primary cellular origins, and temporal expression profiles.

**Table 2:** The role of IL-6.

Cytokine	Main source	Express the sequence of time	Mechanism of action		Quoted
IL-6	Macrophages, fibroblasts, activated Schwann cells	The expression increases within 3 hours after injury, significantly rises within 2 to 5 days, and persists for at least 21 days.	Intensify inflammatory response	Drives macrophage polarization toward the M1 phenotype, exacerbating inflammation.	(32, 33, 38)
				Activates the STAT3 pathway, intensifying neuroinflammation.	(34)
			Enhanced pain signal	Activates the JAK/STAT transduction pathway, leading to mechanical allodynia and thermal hyperalgesia.	(35)
				Mediates bilateral and systemic transmission of inflammatory signals, driving widespread neuroinflammation and pain.	(35)
			Inhibit nerve regeneration	Interferes with Schwann cell function, disrupting axonal integrity.	(36)
				Promotes fibrosis.	(37)
			Bidirectional regulatory effect	Early stage: Recruits macrophages to collaborate with Schwann cells in clearing axonal/myelin debris.	(38, 39)
				Late stage: Collaborates with macrophages to activate the STAT3/STAT6 pathways, secreting enzymes (such as CTSB) to degrade the extracellular matrix (ECM), thereby inhibiting axonal regeneration.	(40)

Summary of the regulatory function of IL-6 in PNI, emphasizing the somatic origin and spatiotemporal expression dynamics mediated by cytokines and secreting cells.

**Table 3:** The role of IL-17.

Cytokine	Main source	Express the sequence of time	Mechanism of action		Quoted
IL-17	Helper T cell 17, neutrophils, mast cells, natural killer (NK) cells	It increases 3 to 7 days after injury and remains highly expressed during the chronic phase	Recruitment of immune cells	Induces the secretion of chemokines, participating in macrophage recruitment.	(14)
			Inhibit nerve regeneration	Upregulates MMP-9 activity, exacerbates inflammatory responses, and indirectly inhibits the myelin regeneration function of Schwann cells.	(43)
				Promotes fibroblast proliferation, leading to endoneurial fibrosis.	(44)
				Facilitates spinal cord (SC)-mediated demyelination and reduces stem cell-mediated myelination.	(45)
			Increased pain	Activates spinal astrocytes and microglia, triggering pro-inflammatory pathways and exacerbating pain.	(41)
				Activates inflammatory cascades by stimulating chemokine ligands, alters neuronal sensitivity, and mediates inflammatory pain.	(43)

The regulatory role of IL-17 in peripheral nerve injury (PNI) was briefly summarized, with emphasis on the participation of cytokines, their main sources and expression times.

### 3.2 The role of anti-inflammatory interleukins

Anti-inflammatory interleukins, such as IL-4, IL-10, and IL-13, play a critical role in enhancing the repair microenvironment following peripheral nerve injury. These cytokines exert their effects by inhibiting pro-inflammatory responses, promoting Schwann cell survival, facilitating myelin regeneration, and modulating immune cell activity. Wallerian degeneration is a series of degenerative changes that occur in the distal axon segment after peripheral nerve injury. By coordinating axonal degeneration, cell activation and inflammatory regulation, it attempts to clear obstacles and support regeneration. It was first described by Augustus Waller in 1850 (46). Among them, IL-4, IL-10 and IL-13 play a key role in the later stage of Wallerian degeneration, not only assisting Schwann cells in completing the transformation from the repair phenotype to the myelin formation phenotype. It also creates favorable conditions for axon regeneration by inhibiting excessive inflammatory responses. Notably, IL-13 and IL-4 exhibit a dual role in extracellular matrix remodeling: moderate signaling facilitates functional repair, while excessive activation may lead to pathological fibrosis (47).This suggests that we should pay attention to the fact that anti-inflammatory interleukins such as IL-4 and IL-13 are also a “double-edged sword” in the repair of peripheral nerve injury.

#### 3.2.1 Interleukin-4

Interleukin-4 (IL-4) is an anti-inflammatory cytokine produced by various immune cells (48). In response to injury, IL-4 is primarily derived from basophils and mast cells; however, other innate immune cells, such as natural killer (NK) T cells and neutrophils, can also express IL-4. Its expression is upregulated within 30 minutes to 24 hours following injury, coinciding with the peak of the inflammatory response (49). IL-4 plays a critical role in nerve injury and regeneration.

##### 3.2.1.1 Promote macrophage polarization

IL-4 plays a critical role in the regulation of Schwann cells (SCs) through both direct and indirect pathways, serving as a key factor essential for optimal regeneration and functional recovery following nerve injury (50). Indirectly, IL-4 modulates Schwann cells by promoting macrophage polarization toward the M2 phenotype (51), thereby exerting anti-inflammatory effects and enhancing tissue repair (47). Directly, IL-4 interacts with Schwann cell receptors, stimulating their migration, alignment, and myelination, which in turn facilitates nerve regeneration (49). This dual action of IL-4 enhances Schwann cell infiltration, as well as axonal regeneration and maturation, significantly increasing the ratio of pro-healing M2 macrophages to pro-inflammatory M1 macrophages at the injury site, thereby promoting successful nerve regeneration (52).

##### 3.2.1.2 Anti-inflammatory effect

Studies have demonstrated that administering IL-4, either concurrently with or shortly after inflammatory stimuli, exerts significant anti-inflammatory effects in macrophages. IL-4 can downregulate the production of pro-inflammatory cytokines, such as TNF and IL-1β, and reduce the release of factors that enhance neuronal excitability, thereby alleviating tactile allodynia (53). Additionally, IL-4 promotes the secretion of anti-inflammatory factors by upregulating the expression of cytokines such as IL-10 and TGF-β, thereby enhancing the immunosuppressive functions of macrophages (54).

##### 3.2.1.3 Neuroprotection and regeneration

Neutralizing endogenous IL-4 significantly enhances neuronal survival rates (55), while exogenous stimulation of the IL-4 signaling pathway promotes neuronal survival following injury. Notably, cells involved in nerve regeneration, including neurons, can respond to IL-4 signaling either directly or indirectly via other immune cells, thereby facilitating regeneration. IL-4 indirectly regulates Schwann cell function by promoting macrophage M2 polarization, while also directly acting on Schwann cell receptors to enhance their migration, alignment, and myelination, thus driving nerve regeneration (49).

#### 3.2.2 Interleukin-10

IL-10 is a potent anti-inflammatory cytokine secreted by both immune and glial cells, playing a crucial role in regulating various anti-inflammatory processes (56). It is predominantly released by infiltrating macrophages, although activated Schwann cells and other immune cells may also contribute to IL-10 production (57). The expression of IL-10 begins to increase significantly in the distal nerve stump four days post-injury, peaks on day 7, and remains elevated throughout the regeneration process (58).

##### 3.2.2.1 Inhibit inflammatory responses

IL-10 is a crucial regulator of inflammatory resolution. It plays a pivotal role in controlling both the early influx of immune cells to the injured nerve and the subsequent efflux of macrophages (59). Following nerve injury, IL-10 levels at the injury site increase significantly. Through the sustained and direct release of IL-10, it promotes the transformation of pro-inflammatory macrophages into an anti-inflammatory phenotype, thereby accelerating the resolution of inflammation and facilitating the recovery process after peripheral nerve injury (59).

##### 3.2.2.2 Promote nerve regeneration

In terms of nerve regeneration, interleukin-10 (IL-10) facilitates the transition of Schwann cells from a “quiescent myelin-maintenance state” to a “migratory/secretory repair state” by activating the STAT3 signaling pathway. This activation significantly enhances Schwann cell migration and the secretion of neurotrophic factors, such as NGF and BDNF, thus providing a cellular foundation for nerve regeneration (60). Additionally, IL-10 modulates macrophage polarization, driving their conversion into a pro-repair phenotype. These macrophages effectively reduce cell apoptosis and promote tissue repair by synthesizing extracellular matrix components and releasing growth factors, thereby supporting axonal regeneration and functional recovery following nerve injury (61).

##### 3.2.2.3 Pain relief

As an anti-inflammatory cytokine, IL-10 plays a crucial protective role in neuropathic pain. It exerts its effects by modulating the activity of nuclear factor-kappa B (NFκB) or by inhibiting the release of pro-inflammatory factors, such as IL-1β and tumor necrosis factor-alpha. This action significantly dampens the inflammatory cascade, thereby reducing pain development and providing analgesic effects (62). Furthermore, cellular injury triggers the activation of CD8+ T cells, which regulate macrophage polarization via TIM3 signaling, indirectly promoting IL-10 secretion. This modulation contributes to a decrease in the excitability and spontaneous activity of peripheral sensory neurons, thereby attenuating pain signal transmission and facilitating the resolution of endogenous pain (63).

#### 3.2.3 Interleukin-13

Interleukin 13 (IL-13) is a well-established anti-inflammatory cytokine that promotes cellular repair and regeneration in response to inflammatory conditions. It plays a crucial role in neuroinflammation within neuropathological settings (64). As a Th2 effector cytokine, IL-13 is secreted by immune cells, including macrophages and Th2 cells. After nerve injury, IL-13 expression is associated with the upregulation of M2-associated genes, particularly becoming more pronounced during the macrophage infiltration phase, which begins around day 3 post-injury (65).

##### 3.2.3.1 Regulate the cell repair microenvironment

IL-13 plays a pivotal role in establishing a tissue-repairing environment by polarizing macrophages toward the M2 phenotype. Sharing receptor subunits with IL-4, IL-13 is considered a prototypical “anti-inflammatory” cytokine due to its ability to suppress type 1 inflammation without inducing immunosuppression. This action promotes M2 macrophage differentiation and initiates a robust type 2 inflammatory response (66). Furthermore, IL-13 sustains the polarized M2 state, which in turn facilitates the secretion of neurotrophic factors, such as BDNF and NGF, along with polyamine synthesis. These processes contribute to the creation of a favorable microenvironment for axonal regeneration, supporting axonal regrowth and functional recovery. This provides a crucial molecular foundation for nerve injury repair (67).

##### 3.2.3.2 Reduce neuronal excitement

After nerve injury, IL-13 induces macrophages to produce IL-10, which in turn reduces the excitability and spontaneous activity of peripheral sensory neurons, facilitating the resolution of peripheral nerve injury (63). Additionally, IL-13 inhibits the polarization of pro-inflammatory M1 macrophages, resulting in a decreased production of neuronal excitability-enhancing factors such as IL-1β and CCL3. This effect indirectly reduces abnormal neuronal excitability and alleviates tactile allodynia (68).

##### 3.2.3.3 Extracellular matrix remodeling

Both IL-13 and IL-4 signal through the type II IL-4 receptor, with IL-13 directly modulating extracellular matrix synthesis and remodeling in fibroblasts via the IL-4Rα/IL-13Rα1-JAK-STAT6 pathway. Together, they synergistically promote fibroblast activation, enhancing the synthesis and deposition of collagen and fibronectin, which contributes to scar formation or regeneration and provides structural support for axonal regrowth (69).

**Figure 3:**
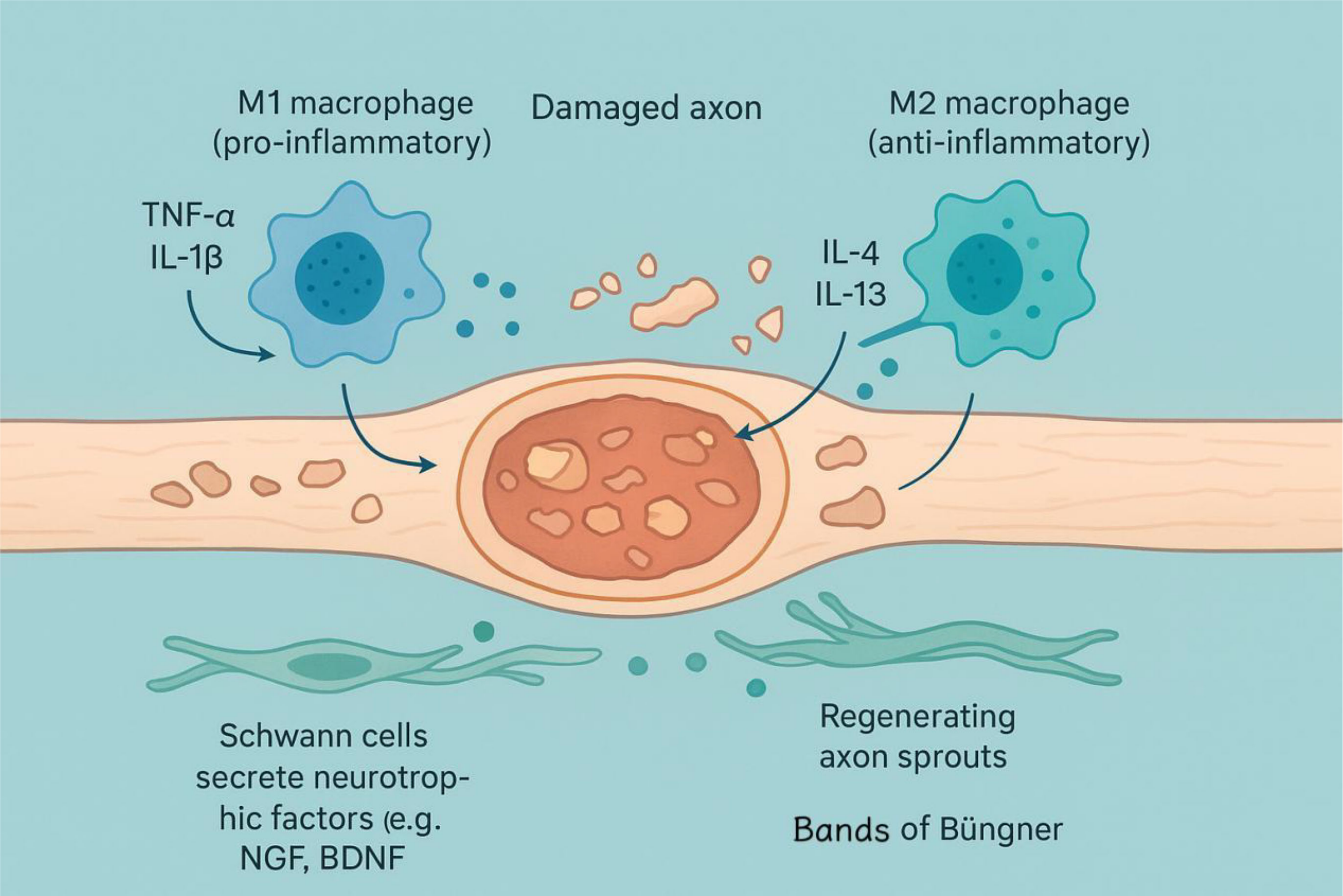
After nerve injury occurs, pro-inflammatory M1-type macrophages are activated, releasing inflammatory factors such as TNF-α and IL-1β. Anti-inflammatory M2-type macrophages secrete cytokines such as IL-4 and IL-13, which help regulate inflammatory responses and promote repair. Meanwhile, Schwann cells are activated and secrete neurotrophic factors such as NGF and BDNF, providing support for axon regeneration. Ultimately, the regenerated axon buds grow forward along the Bungner band composed of Schwann cells, completing the nerve repair process. The diagram was drawn by the authors using Bio Render.

The demonstrated capabilities of anti-inflammatory interleukins (IL-4, IL-10, IL-13) in modulating the immune microenvironment, promoting repair, and alleviating pain underscore their potential as therapeutic agents to actively enhance nerve regeneration and functional recovery following injury. To more clearly explain the source, expression time series and mechanism of action of anti-inflammatory interleukins, the summary is shown in **Tables 4**–**6**. The mechanism of action is shown in **Figure 3**.

**Table 4:** The role of IL-4.

Cytokine	Main source	Express the sequence of time	Mechanism of action		Quoted
IL-4	Basophilic granulocytes, mast cells, NK T cells and neutrophils	The expression is upregulated within 30 minutes to 24 hours after injury, accompanied by a peak of inflammatory response.	Promote macrophage polarization	Promoting the differentiation of M2-type macrophages	(47, 50, 51)
			Anti-inflammatory effect	inhibiting the release of pro-inflammatory factors (TNF-α, IL-1β) to alleviate neuroinflammation	(49, 52)
			Neuroprotection and regeneration	Reducing neuronal apoptosis	(49, 55)

A brief overview of the regulatory role of IL-4 in peripheral nerve injury (PNI) is provided, with a focus on cytokines, major sources, and expression time.

**Table 5:** The role of IL-10.

Cytokine	Main source	Express the sequence of time	Mechanism of action		Quoted
IL-10	Schwann cells, infiltrating macrophages and other immune cells	It begins to increase 4 days after the injury, reaches its peak on the 7th day, and continues until the repair stage	Inhibit inflammatory responses	Directly inhibit the expression of pro-inflammatory cytokines and chemokines.	(59)
			Promote nerve regeneration	Stimulate Schwann cell migration and neurotrophic factor secretion.	(60, 61)
			Pain relief	Suppress pro-inflammatory factors, neuronal excitability, and spontaneous activity to alleviate neuropathic pain.	(62, 63)

A brief overview of the regulatory role of IL-10 in peripheral nerve injury (PNI), with a focus on its interaction with cytokines, main sources, and expression time.

**Table 6:** The role of IL-13.

Cytokine	Main source	Express the sequence of time	Mechanism of action		Quoted
IL-13	Immune cells (such as macrophages, Th2 cells	It gradually strengthens from 3 days after the injury	Regulate the microenvironment of cell repair	Stimulates macrophages to produce IL-10, thereby reducing the excitability and spontaneous activity of peripheral sensory neurons.	(66, 67)
			Reduce neuronal excitement	Shares receptor subunits with IL-4 and synergistically promotes M2 macrophage differentiation.	(63, 68)
			Extracellular matrix remodeling	Indirectly provides nutritional support for axonal regeneration by polarizing macrophages.	(69)

It summarizes the regulatory role of IL-13 in peripheral nerve injury (PNI), with a focus on the participation, main sources and expression time of cytokines.

## 4 The therapeutic potential of interleukins in peripheral nerve injury

### 4.1 Application of interleukin inhibitors and agonists in nerve injury

Research on interleukin-targeted therapies for peripheral nerve injury is still in the exploratory phase; however, their potential in modulating inflammation, regulating the immune microenvironment, and promoting nerve repair is increasingly gaining attention.

#### 4.1.1 Application of interleukin inhibitors in nerve injury

Inhibiting pro-inflammatory interleukins, such as IL-1, IL-6, and IL-17, can mitigate the early excessive inflammatory response and alleviate secondary nerve injury.

IL-1 inhibitors, such as anakinra, are competitive antagonists of the IL-1 receptor and function as anti-inflammatory agents. These inhibitors regulate the biological activity of IL-1β by blocking signal transduction, thereby preventing cytokine release and processes associated with antioxidant activity, which in turn helps alleviate neuropathic pain (70). For example, in studies investigating vincristine-induced peripheral nerve injury, vincristine was shown to induce macrophage release of IL-1β through the activation of the NLRP3 inflammasome. Anakinra specifically inhibits this process, reducing the inflammatory cascade linked to nerve injury and preventing vincristine-induced neuropathy (71).

IL-6 inhibitors, such as tocilizumab, are monoclonal antibodies that modulate immune responses to various stimuli by blocking the action of interleukin-6 (IL-6) (72). Tocilizumab neutralizes IL-6R, thereby inhibiting both “classic signaling” and “trans-signaling” pathways of IL-6, which in turn suppresses neuroinflammatory responses. Furthermore, in chemotherapy-induced peripheral neuropathy (CIPN), tocilizumab may alleviate pain symptoms by attenuating IL-6-related inflammatory responses (73).

IL-17A inhibitors, such as secukinumab, are fully human anti-IL-17A (IL-17A) monoclonal antibodies that selectively target and neutralize IL-17A (74). By blocking the binding of IL-17A to its receptors (IL-17RA/C), these inhibitors disrupt downstream inflammatory pathways, including NF-κB and MAPK signaling, thereby reducing the release of pro-inflammatory cytokines (e.g., TNF-α, IL-6) and chemokines. This leads to a decrease in neuroinflammation and alleviation of pain symptoms, offering a promising therapeutic target for chronic pain associated with peripheral nerve injury (43).

This inhibitory method directly eliminates harmful mechanisms, aiming to reduce inflammatory amplification, prevent Schwann cell dysfunction and pyroapoptosis, reduce fibrosis, and alleviate neuropathic pain.

#### 4.1.2 Application of interleukin agonists in nerve injury

Anti-inflammatory and tissue-repair-promoting interleukins, such as IL-4, IL-10, and IL-13, can enhance anti-inflammatory responses, promote macrophage polarization toward the M2 phenotype, and accelerate nerve repair. For instance, IL-4/IL-13 agonists facilitate M2 macrophage polarization. These interleukins stimulate the secretion of anti-inflammatory factors (e.g., TGF-β and IL-10) and neurotrophic factors, thereby enhancing Schwann cell function and axonal regeneration. Simultaneously, through the activation of the JAK-STAT6 pathway, IL-4/IL-13 agonists inhibit the production of pro-inflammatory cytokines (such as TNF-α and IL-1β), promote M2 macrophage polarization, and enhance their ability to clear cellular debris and secrete neurotrophic factors (such as IGF-1 and PDGF), thus optimizing the microenvironment for nerve repair. Additionally, IL-13 induces IL-10 production, which drives the transition of inflammatory M1 macrophages to reparative M2 macrophages, thereby alleviating neuropathic pain (e.g., chemotherapy-induced neuropathy) (75). IL-10 agonists can directly suppress pro-inflammatory cytokines. Upon binding to its receptors (IL-10R1/R2), IL-10 activates the JAK-STAT3 signaling pathway, inhibiting NF-κB activity. This leads to a reduction in the production of pro-inflammatory cytokines (such as IL-1β and TNF-α) and chemokines (such as CCL2), while upregulating Schwann cell activity (76).

Agonists can enhance the beneficial processes facilitated by anti-inflammatory factors, thereby strengthening endogenous repair mechanisms, shifting the immune balance towards a pro-regenerative state, and actively supporting Schwann cell function and axon growth.

### 4.2 Combined application of interleukins with other therapeutic strategies

While targeting interleukin pathways alone holds promise, the complexity of PNI suggests that combining interleukin-based strategies with other established or emerging therapeutic modalities may yield synergistic effects, leading to more robust and comprehensive nerve repair.

#### 4.2.1 Interleukin combined with neurotrophic factor

Certain interleukins inherently possess neurotrophic effects, while neurotrophic factors, such as NGF, BDNF, GDNF, and NT-3, directly promote neuronal survival and axonal growth. The combination of these factors can result in additive or synergistic effects.

IL-6 + NGF/BDNF: IL-6, secreted by activated macrophages and monocytes, plays a pivotal role in the acute-phase response following nerve injury, which is characterized by symptoms such as fever, changes in sleep, and alterations in appetite. IL-6 enhances the expression of BDNF and its receptor in the spinal cord, thereby activating neurotrophic factor signaling pathways. This promotes neuronal survival and axonal regeneration. Studies have shown that inhibiting IL-6-mediated inflammatory responses while simultaneously enhancing the neurotrophic support provided by BDNF and GDNF can significantly improve functional recovery and alleviate pain following nerve injury (77).

IL-10 + GDNF: IL-10, synthesized by microglia and astrocytes, exerts a suppressive effect on the secretion of pro-inflammatory cytokines, such as TNF-α, by microglia. GDNF, a member of the neurotrophic factor family, promotes neuronal survival and differentiation. The combined upregulation of IL-10 and GDNF enhances both their anti-inflammatory and neurotrophic effects. This synergistic approach has been shown to effectively mitigate pain behaviors induced by nerve injury (78).

#### 4.2.2 Interleukin combined with cell therapy

Research indicates that Schwann cells, stem cells, and other cell types have the ability to secrete a variety of neurotrophic factors and cytokines. When these cells are treated with specific interleukins or co-delivered with interleukins, their survival, migration, and nerve repair capabilities can be significantly enhanced.

Stem Cells and IL-10:Studies have demonstrated that interleukin-10 (IL-10) selectively promotes the migration and cytokine secretion programs of mesenchymal stem cells (MSCs), thereby augmenting their anti-inflammatory therapeutic effects. MSCs regulate local immune responses by upregulating the expression of anti-inflammatory cytokines. The transient but significant secretion of human IL-10 by MSCs has been shown to facilitate the repair of injured spinal cords (79).

Schwann Cells and IL-1Ra: IL-1 receptor antagonist (IL-1Ra) inhibits macrophage activation and T-cell costimulatory signals by neutralizing the pro-inflammatory activity of IL-1, thereby reducing neuroinflammation and demyelination. Schwann cells (SCs) regulate the IL-1/IL-1Ra balance to limit excessive immune responses during the early stages of inflammation, maintaining immune homeostasis at the site of injury, which are primary sites of macrophage invasion. This regulatory balance creates a favorable microenvironment for axonal regeneration and myelin repair (80).

#### 4.2.3 Interleukin combined with electrical stimulation

Electrical stimulation enhances peripheral nerve repair by modulating the expression of interleukins, thereby optimizing the post-injury inflammatory microenvironment. It acts synergistically with neurotrophic factors and regeneration-associated genes (RAGs). Electrical stimulation promotes the transition of macrophages to an anti-inflammatory phenotype, thereby increasing the secretion of anti-inflammatory cytokines (e.g., IL-10) and inhibiting the release of pro-inflammatory factors (e.g., TNF-α, IL-1β). This modulation reduces neurotoxic damage. Studies have demonstrated that electrical stimulation, in combination with IL-10, can synergistically upregulate RAGs and cytoskeletal proteins (e.g., GAP-43) via the PI3K-Akt and MAPK signaling pathways, thus accelerating axonal regeneration (81).

#### 4.2.4 Interleukin combined with gene therapy

The use of viral or non-viral vectors to overexpress therapeutic interleukins or their receptors locally at the injury site, or in transplanted cells, offers a promising approach for the co-delivery of vectors encoding neurotrophic factors or other repair-related genes. For instance, AAV-IL-10 combined with AAV-GDNF utilizes adeno-associated virus (AAV) or lentiviral vectors to introduce the IL-10 gene into target cells, such as Schwann cells or sensory/motor neurons. This enhances IL-10 expression, thereby promoting anti-inflammatory and neuroprotective effects. Furthermore, the development of regulated gene therapy systems, such as the fusion of the Epstein-Barr virus Gly-Ala repeat domain (GAr) with a transcriptional activator (TA), reduces immunogenicity and enables precise “on/off” control of gene expression (82).

**Figure 4:**
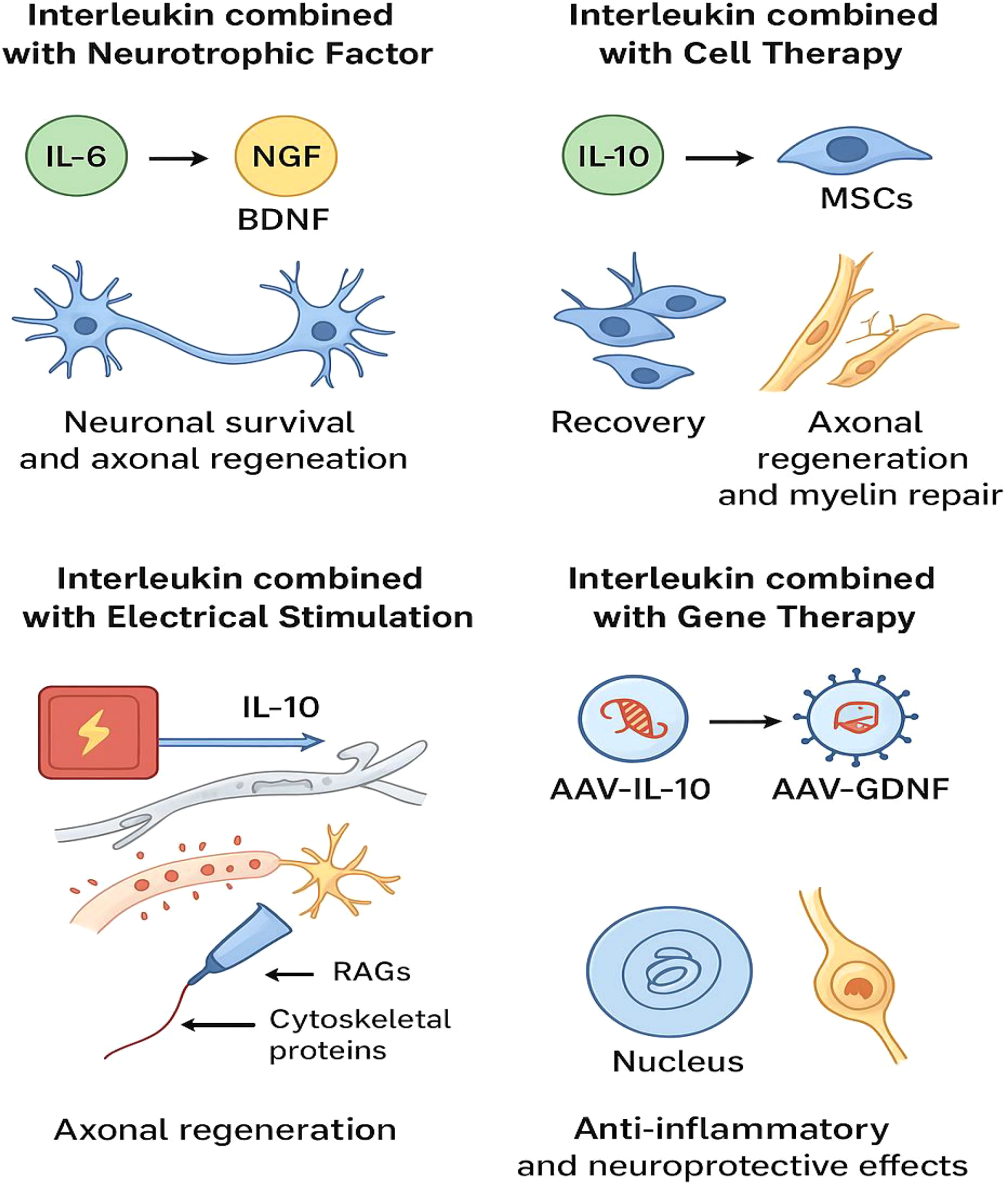
This schematic diagram summarizes four combined treatment strategies centered on interleukin (IL), aiming to promote nerve repair and regeneration. When IL-6 binds to neurotrophic factors such as NGF and BDNF, it can support neuronal survival and axonal regeneration. IL-10 combined with electrical stimulation can upregulate the expression of regeneration-related genes (RAGs) and cytoskeletal proteins, thereby promoting axon regeneration; IL-10 combined with cell therapy (such as mesenchymal stem cells, MSCs) can improve functional recovery, axon regeneration and myelin repair. Through gene therapy methods (such as using AAV vectors to deliver IL-10 and GDNF), dual effects of anti-inflammation and neuroprotection can be achieved, promoting nerve repair in a synergistic manner. The diagram was drawn by the authors using Bio Render.

To more clearly demonstrate the combined application and functions of interleukins with other therapeutic strategies, please refer to **Table 7** for details. The mechanism of action is shown in **Figure 4**.

**Table 7:** The combined application of interleukins with other therapeutic strategies.

Treatment methods	Specific application	Function	Quoted
Combined with neurotrophic factors	IL-6 + NGF/BDNF	Promote neuronal survival and axonal regeneration.	(77)
		Improve functional recovery and pain relief after nerve injury.	
	IL-10 + GDNF	Promote neuronal survival and differentiation.	(78)
		Suppress pain behaviors induced by nerve injury.	
Combined with cell therapy	IL-10+ stem cells	Promote the repair of injured spinal cords.	(79)
	IL-1ra+ Schwann cells	Maintain immune homeostasis and regulate the regenerative microenvironment.	(80)
Combined with physical therapy/electrical stimulation	Electrical stimulation +IL-10	Reduce neurotoxic damage.	(81)

Briefly describe the application of interleukin combined with various therapeutic strategies in the treatment of peripheral nerve injury. Including the application of interleukin combined with neurotrophic factor, cell therapy, physical therapy/electrical stimulation.

## 5 Conclusion

The repair of peripheral nerve injury (PNI) is closely linked to an imbalanced immune microenvironment. As key mediators of immune regulation, the interleukin (IL) family plays a pivotal role in the progression of PNI. Following peripheral nerve injury, local tissues quickly initiate an inflammatory response to clear damage debris and promote repair. Pro-inflammatory interleukins, including IL-1, IL-6, and IL-17, are significantly elevated and dominate the early phase of nerve injury. As the inflammation subsides, anti-inflammatory interleukins, such as IL-4, IL-10, and IL-13, gradually become more prominent, limiting tissue damage. These interleukins work synergistically to optimize the repair microenvironment through distinct signaling pathways, thereby promoting nerve regeneration.

Interleukins play a crucial role in the treatment and neural repair process following PNI. Pro-inflammatory interleukins increase rapidly after nerve injury, with IL-1, IL-6, and IL-17 contributing to nerve damage through multiple mechanisms. These include amplifying inflammatory responses, inhibiting nerve regeneration, and exacerbating pain, ultimately suppressing neural repair and regeneration. In contrast, anti-inflammatory interleukins inhibit inflammatory responses, promote Schwann cell survival, stimulate myelin regeneration, alleviate pain, and facilitate extracellular matrix remodeling during nerve repair. These actions improve the repair microenvironment after PNI and enhance the regeneration of damaged nerves. Of course, it cannot be ignored that interleukins exhibit a time-dependent bidirectional effect in the repair of peripheral nerve injury, such as factors like IL-1β, IL-6, and IL-17. During their chronic and excessive expression, they can impede regeneration and intensify pain, and are often necessary to initiate the repair process in the early stage of injury. However, equally, Anti-inflammatory factors represented by IL-4, IL-10 and IL-13, while promoting repair, also have potential risks such as inducing fibrosis. Modern medical approaches utilize interleukins in combination with various strategies for PNI treatment. These include the use of interleukin inhibitors, such as IL-1 inhibitors (e.g., anakinra), IL-6 inhibitors (e.g., tocilizumab), and IL-17A inhibitors (e.g., secukinumab), as well as interleukin agonists. Additionally, interleukins are combined with neurotrophic factors to enhance neuronal survival and axonal growth, or with cell therapy to improve the survival, migration, and neural repair capacity of transplanted cells via interleukin treatment. Physical therapy modalities, such as electrical stimulation, are also employed to optimize the inflammatory microenvironment following nerve injury, working synergistically with neurotrophic factors to promote peripheral nerve repair. Moreover, interleukins are combined with gene therapy (utilizing viral or non-viral vectors) to overexpress therapeutic interleukins or their receptors locally at the injury site or in transplanted cells, enabling long-term and stable expression.

However, the application of interleukins in the treatment of PNI is not without its contradictions. Pro-inflammatory factors can exacerbate inflammation, induce pain, and inhibit regeneration in the early stages of injury. While anti-inflammatory factors promote repair, challenges remain, including low delivery efficiency and the risk of fibrosis upon excessive activation. Despite the advancement of novel approaches in interleukin-based therapies for PNI, significant bottlenecks remain, warranting ongoing exploration. A deeper understanding of the mechanisms underlying interleukin-based therapies will provide a theoretical foundation for future research. This includes strategies such as localized release of agonists via nanocarriers, the use of biomarkers to guide the administration of IL inhibitors or agonists, and the synergistic activation of regenerative pathways through electrical stimulation or neurotrophic factors. These innovations could significantly expand the potential and diversity of interleukin-based treatments for PNI-related diseases.

## References

[1] Guo XJ Zhang B Wu SH Zhang L Wang Y . The role of the PI3K/Akt signaling pathway in peripheral nerve injury and the research progress of traditional Chinese medicine intervention. Chin J Comp Med. (2024) 34:116–25.

[2] Ye XP Huang HY . Clinical observation of Yiqi Tongbi decoction fumigation and washing in the treatment of Qi deficiency and blood stasis type diabetic peripheral neuropathy. Chin J Integr Med Sci. (2025) 32:190–2.

[3] Hussain G Wang J Rasul A Anwar H Qasim M Zafar S . Current status of therapeutic approaches against peripheral nerve injuries: A detailed story from injury to recovery. Int J Biol Sci. (2020) 16:116–34. doi: 10.7150/ijbs.35653, PMID: 31892850 PMC6930373

[4] Lopes B Sousa P Alvites R Branquinho M Sousa AC Mendonça C . Peripheral nerve injury treatments and advances: one health perspective. Int J Mol Sci. (2022) 23:918. doi: 10.3390/ijms23020918, PMID: 35055104 PMC8779751

[5] Wang ML Rivlin M Graham JG Beredjiklian PK . Peripheral nerve injury, scarring, and recovery. Connective Tissue Res. (2019) 60:3–9. doi: 10.1080/03008207.2018.1489381, PMID: 30187777

[6] Modrak M Talukder MH Gurgenashvili K Noble M Elfar JC . Peripheral nerve injury and myelination: Potential therapeutic strategies. J Neurosci Res. (2020) 98:780–95. doi: 10.1002/jnr.24538, PMID: 31608497 PMC7072007

[7] Liu X Duan X . Mechanisms and treatments of peripheral nerve injury. Ann Plast Surg. (2023) 91:313–8. doi: 10.1097/SAP.0000000000003480, PMID: 36880740

[8] Brocker C Thompson D Matsumoto A Nebert DW Vasiliou V . Evolutionary divergence and functions of the human interleukin (IL) gene family. Hum Genomics. (2010) 5:30–55. doi: 10.1186/1479-7364-5-1-30, PMID: 21106488 PMC3390169

[9] Bernardi S Marcuzzi A Piscianz E Tommasini A Fabris B . The complex interplay between lipids, immune system and interleukins in cardio-metabolic diseases. Int J Mol Sci. (2018) 19:4058. doi: 10.3390/ijms19124058, PMID: 30558209 PMC6321433

[10] Dawalibi A Alosaimi AA Mohammad KS . Balancing the scales: the dual role of interleukins in bone metastatic microenvironments. Int J Mol Sci. (2024) 25:8163. doi: 10.3390/ijms25158163, PMID: 39125732 PMC11311339

[11] Liu P Peng J Han GH Ding X Wei S Gao G . Role of macrophages in peripheral nerve injury and repair. Neural regeneration Res. (2019) 14:1335–42. doi: 10.4103/1673-5374.253510, PMID: 30964051 PMC6524518

[12] Pottorf TS Rotterman TM McCallum WM Haley-Johnson ZA Alvarez FJ . The role of microglia in neuroinflammation of the spinal cord after peripheral nerve injury. Cells. (2022) 11:2083. doi: 10.3390/cells11132083, PMID: 35805167 PMC9265514

[13] Camara-Lemarroy CR Gonzalez-Moreno EI Guzman-de la Garza FJ Fernandez-Garza NE . Arachidonic acid derivatives and their role in peripheral nerve degeneration and regeneration. ScientificWorldJournal. (2012) 2012:168953. doi: 10.1100/2012/168953, PMID: 22997489 PMC3446639

[14] Huang Y Wu L Zhao Y Guo J Li R Ma S . Schwann cell promotes macrophage recruitment through IL-17B/IL-17RB pathway in injured peripheral nerves. Cell Rep. (2024) 43:113753. doi: 10.1016/j.celrep.2024.113753, PMID: 38341853

[15] Mai CL Wei X Gui WS Xu YN Zhang J Lin ZJ . Differential regulation of GSK-3β in spinal dorsal horn and in hippocampus mediated by interleukin-1beta contributes to pain hypersensitivity and memory deficits following peripheral nerve injury. Mol Pain. (2019) 15:1744806919826789. doi: 10.1177/1744806919826789, PMID: 30632435 PMC6378430

[16] Li X Guan Y Li C Zhang T Meng F Zhang J . Immunomodulatory effects of mesenchymal stem cells in peripheral nerve injury. Stem Cell Res Ther. (2022) 13:18. doi: 10.1186/s13287-021-02690-2, PMID: 35033187 PMC8760713

[17] Fischer S Weishaupt A Troppmair J Martini R . Increase of MCP-1 (CCL2) in myelin mutant Schwann cells is mediated by MEK-ERK signaling pathway. Glia. (2008) 56:836–43. doi: 10.1002/glia.20657, PMID: 18383340

[18] Wang F Zhao C Jing Z Wang Q Li M Lu B . The dual roles of chemokines in peripheral nerve injury and repair. Inflammation regeneration. (2025) 45:11. doi: 10.1186/s41232-025-00375-4, PMID: 40217284 PMC11987372

[19] Molnár K Nógrádi B Kristóf R Mészáros Á Pajer K Siklós L . Motoneuronal inflammasome activation triggers excessive neuroinflammation and impedes regeneration after sciatic nerve injury. J Neuroinflamm. (2022) 19:68. doi: 10.1186/s12974-022-02427-9, PMID: 35305649 PMC8934511

[20] Wang J Chen P Han G Zhou Y Xiang X Bian M . Rab32 facilitates Schwann cell pyroptosis in rats following peripheral nerve injury by elevating ROS levels. J Trans Med. (2024) 22:194. doi: 10.1186/s12967-024-04999-x, PMID: 38388913 PMC10885539

[21] Wüst HM Wegener A Fröb F Hartwig AC Wegwitz F Kari V . Egr2-guided histone H2B monoubiquitination is required for peripheral nervous system myelination. Nucleic Acids Res. (2020) 48:8959–76. doi: 10.1093/nar/gkaa606, PMID: 32672815 PMC7498331

[22] Del Rey A Yau HJ Randolf A Centeno MV Wildmann J Martina M . Chronic neuropathic pain-like behavior correlates with IL-1β expression and disrupts cytokine interactions in the hippocampus. Pain. (2011) 152:2827–35. doi: 10.1016/j.pain.2011.09.013, PMID: 22033365 PMC3215892

[23] Qu WR Zhu Z Liu J Song DB Tian H Chen BP . Interaction between Schwann cells and other cells during repair of peripheral nerve injury. Neural Regener Res. (2021) 16:93–8. doi: 10.4103/1673-5374.286956, PMID: 32788452 PMC7818858

[24] Wang J Lu S Yuan Y Huang L Bian M Yu J . Inhibition of schwann cell pyroptosis promotes nerve regeneration in peripheral nerve injury in rats. Mediators Inflammation. (2023) 2023:9721375. doi: 10.1155/2023/9721375, PMID: 37144237 PMC10154099

[25] Parish CL Finkelstein DI Tripanichkul W Satoskar AR Drago J Horne MK . The role of interleukin-1, interleukin-6, and glia in inducing growth of neuronal terminal arbors in mice. J Neurosci. (2002) 22:8034–41. doi: 10.1523/JNEUROSCI.22-18-08034.2002, PMID: 12223557 PMC6758077

[26] Zhang J Zhang X Li L Bai L Gao Y Yang Y . Activation of double-stranded RNA-activated protein kinase in the dorsal root ganglia and spinal dorsal horn regulates neuropathic pain following peripheral nerve injury in rats. Neurotherapeutics. (2022) 19:1381–400. doi: 10.1007/s13311-022-01255-2, PMID: 35655111 PMC9587175

[27] Smith PA . Neuropathic pain; what we know and what we should do about it. Front Pain Res (Lausanne Switzerland). (2023) 4:1220034. doi: 10.3389/fpain.2023.1220034, PMID: 37810432 PMC10559888

[28] Boakye PA Tang SJ Smith PA . Mediators of neuropathic pain; focus on spinal microglia, CSF-1, BDNF, CCL21, TNF-α, wnt ligands, and interleukin 1β. Front Pain Res (Lausanne Switzerland). (2021) 2:698157. doi: 10.3389/fpain.2021.698157, PMID: 35295524 PMC8915739

[29] Magyari L Kovesdi E Sarlos P Javorhazy A Sumegi K Melegh B . Interleukin and interleukin receptor gene polymorphisms in inflammatory bowel diseases susceptibility. World J Gastroenterol. (2014) 20:3208–22. doi: 10.3748/wjg.v20.i12.3208, PMID: 24695754 PMC3964393

[30] Fregnan F Muratori L Simões AR Giacobini-Robecchi MG Raimondo S . Role of inflammatory cytokines in peripheral nerve injury☆. Neural regeneration Res. (2012) 7:2259–66., PMID: 25538747 10.3969/j.issn.1673-5374.2012.29.003 PMC4268726

[31] Eliav E Benoliel R Herzberg U Kalladka M Tal M . The role of IL-6 and IL-1β in painful perineural inflammatory neuritis. Brain behavior Immun. (2009) 23:474–84. doi: 10.1016/j.bbi.2009.01.012, PMID: 19486649

[32] Guerrero AR Uchida K Nakajima H Watanabe S Nakamura M Johnson WE . Blockade of interleukin-6 signaling inhibits the classic pathway and promotes an alternative pathway of macrophage activation after spinal cord injury in mice. J Neuroinflamm. (2012) 9:40. doi: 10.1186/1742-2094-9-40, PMID: 22369693 PMC3310810

[33] Fu W . Clinical study on transcutaneous acupoint electroacupuncture for chemotherapy-induced peripheral neuropathy in cancer patients. Med Theory Pract. (2024) 37:4111–3. doi: 10.19381/j.issn.1001-7585.2024.23.054

[34] Liu M Pan J Li X Zhang X Tian F Li M . Interleukin-6 deficiency reduces neuroinflammation by inhibiting the STAT3-cGAS-STING pathway in Alzheimer’s disease mice. J Neuroinflamm. (2024) 21:282. doi: 10.1186/s12974-024-03277-3, PMID: 39487531 PMC11529443

[35] Brázda V Klusáková I Svíženská IH Dubový P . Dynamic response to peripheral nerve injury detected by in *situ* hybridization of IL-6 and its receptor mRNAs in the dorsal root ganglia is not strictly correlated with signs of neuropathic pain. Mol Pain. (2013) 9:1744–8069. doi: 10.1186/1744-8069-9-42, PMID: 23953943 PMC3844395

[36] Nashtahosseini Z Eslami M Paraandavaji E Haraj A Dowlat BF Hosseinzadeh E . Cytokine signaling in diabetic neuropathy: A key player in peripheral nerve damage. Biomedicines. (2025) 13:589. doi: 10.3390/biomedicines13030589, PMID: 40149566 PMC11940495

[37] Mosleh H Hosseini S Hajizadeh N Majdi L Ajdary M Mofarahe ZS . Role of neuropeptides in patients with endometriosis: a literature review. Middle East Fertility Soc J. (2024) 29:49. doi: 10.1186/s43043-024-00207-4

[38] Chen Y Deng H Zhang N . Autophagy-targeting modulation to promote peripheral nerve regeneration. Neural regeneration Res. (2025) 20:1864–82. doi: 10.4103/NRR.NRR-D-23-01948, PMID: 39254547 PMC11691477

[39] Liu C Liu D Zhang X Hui L Zhao L . Nanofibrous polycaprolactone/amniotic membrane facilitates peripheral nerve regeneration by promoting macrophage polarization and regulating inflammatory microenvironment. Int Immunopharmacol. (2023) 121:110507. doi: 10.1016/j.intimp.2023.110507, PMID: 37356125

[40] Zhang S Zhu H Li G Zhu M . Cathepsin B promotes optic nerve axonal regeneration. Neuroreport. (2025) 36:279–89. doi: 10.1097/WNR.0000000000002148, PMID: 40177832 PMC11949221

[41] Gao SJ Liu L Li DY Liu DQ Zhang LQ Wu JY . Interleukin-17: A putative novel pharmacological target for pathological pain. Curr neuropharmacology. (2024) 22:204–16. doi: 10.2174/1570159X21666230811142713, PMID: 37581321 PMC10788884

[42] Noma N Khan J Chen IF Markman S Benoliel R Hadlaq E . Interleukin-17 levels in rat models of nerve damage and neuropathic pain. Neurosci Lett. (2011) 493:86–91. doi: 10.1016/j.neulet.2011.01.079, PMID: 21316418

[43] Jiang X Zhou R Zhang Y Zhu T Li Q Zhang W . Interleukin-17 as a potential therapeutic target for chronic pain. Front Immunol. (2022) 13:999407. doi: 10.3389/fimmu.2022.999407, PMID: 36248896 PMC9556763

[44] Aldali F Deng C Nie M Chen H . Advances in therapies using mesenchymal stem cells and their exosomes for treatment of peripheral nerve injury: state of the art and future perspectives. Neural Regeneration Res. (2025) 20:3151–71. doi: 10.4103/NRR.NRR-D-24-00235, PMID: 39435603 PMC11881730

[45] Stettner M Lohmann B Wolffram K Weinberger JP Dehmel T Hartung HP . Interleukin-17 impedes Schwann cell-mediated myelination. J Neuroinflamm. (2014) 11:63. doi: 10.1186/1742-2094-11-63, PMID: 24678820 PMC3977670

[46] Gaudet AD Popovich PG Ramer MS . Wallerian degeneration: gaining perspective on inflammatory events after peripheral nerve injury. J Neuroinflammation. (2011) 8:110. doi: 10.1186/1742-2094-8-110, PMID: 21878126 PMC3180276

[47] Zigmond RE Echevarria FD . Macrophage biology in the peripheral nervous system after injury. Prog Neurobiol. (2019) 173:102–21. doi: 10.1016/j.pneurobio.2018.12.001, PMID: 30579784 PMC6340791

[48] Zhang Q Zhu W Xu F . The interleukin-4/PPARγ signaling axis promotes oligodendrocyte differentiation and remyelination after brain injury. PLoS Biol. (2019) 17:e3000330. doi: 10.1371/journal.pbio.3000330, PMID: 31226122 PMC6608986

[49] Daines JM Schellhardt L Wood MD . The role of the IL-4 signaling pathway in traumatic nerve injuries. Neurorehabilitation Neural Repair. (2021) 35:431–43. doi: 10.1177/15459683211001026, PMID: 33754913 PMC8122057

[50] Pan D Schellhardt L Acevedo-Cintron JA Hunter D Snyder-Warwick AK Mackinnon SE . IL-4 expressing cells are recruited to nerve after injury and promote regeneration. Exp Neurol. (2022) 347:113909. doi: 10.1016/j.expneurol.2021.113909, PMID: 34717939 PMC8887027

[51] Enam SF Kader SR Bodkin N Lyon JG Calhoun M Azrak C . Evaluation of M2-like macrophage enrichment after diffuse traumatic brain injury through transient interleukin-4 expression from engineered mesenchymal stromal cells. J Neuroinflamm. (2020) 17:197. doi: 10.1186/s12974-020-01860-y, PMID: 32563258 PMC7306141

[52] Wofford KL Shultz RB Burrell JC Cullen DK . Neuroimmune interactions and immunoengineering strategies in peripheral nerve repair. Prog Neurobiol. (2022) 208:102172. doi: 10.1016/j.pneurobio.2021.102172, PMID: 34492307 PMC8712351

[53] Gadani SP Cronk JC Norris GT Kipnis J . IL-4 in the brain: a cytokine to remember. J Immunol (Baltimore Md.: 1950). (2012) 189:4213–9. doi: 10.4049/jimmunol.1202246, PMID: 23087426 PMC3481177

[54] Shi Q Cai X Shi G Lv X Yu J Wang F . Interleukin-4 protects from chemotherapy-induced peripheral neuropathy in mice modal via the stimulation of IL-4/STAT6 signaling. Acta cirurgica Bras. (2018) 33:491–8. doi: 10.1590/s0102-865020180060000003, PMID: 30020310

[55] Jang J Hong A Chung Y Jin B . Interleukin-4 aggravates LPS-induced striatal neurodegeneration *in vivo* via oxidative stress and polarization of microglia/macrophages. Int J Mol Sci. (2022) 23:571. doi: 10.3390/ijms23010571, PMID: 35008995 PMC8745503

[56] Kwilasz AJ Grace PM Serbedzija P Maier SF Watkins LR . The therapeutic potential of interleukin-10 in neuroimmune diseases. Neuropharmacology. (2015) 96:55–69. doi: 10.1016/j.neuropharm.2014.10.020, PMID: 25446571 PMC5144739

[57] Dubový P Klusáková I Hradilová Svíženská I . Inflammatory profiling of Schwann cells in contact with growing axons distal to nerve injury. BioMed Res international. (2014) 2014:691041. doi: 10.1155/2014/691041, PMID: 24877128 PMC4022316

[58] Woodley PK Min Q Li Y Mulvey NF Parkinson DB Dun XP . Distinct VIP and PACAP functions in the distal nerve stump during peripheral nerve regeneration. Front Neurosci. (2019) 13:1326. doi: 10.3389/fnins.2019.01326, PMID: 31920495 PMC6920234

[59] Siqueira Mietto B Kroner A Girolami EI Santos-Nogueira E Zhang J David S . Role of IL-10 in resolution of inflammation and functional recovery after peripheral nerve injury. J neuroscience: Off J Soc Neurosci. (2015) 35:16431–42. doi: 10.1523/JNEUROSCI.2119-15.2015, PMID: 26674868 PMC6605511

[60] Rao M Nelms BD Dong L Salinas-Rios V Rutlin M Gershon MD . Enteric glia express proteolipid protein 1 and are a transcriptionally unique population of glia in the mammalian nervous system. Glia. (2015) 63:2040–57. doi: 10.1002/glia.22876, PMID: 26119414 PMC4695324

[61] Hellenbrand DJ Reichl KA Travis BJ Filipp ME Khalil AS Pulito DJ . Sustained interleukin-10 delivery reduces inflammation and improves motor function after spinal cord injury. J Neuroinflamm. (2019) 16:93. doi: 10.1186/s12974-019-1479-3, PMID: 31039819 PMC6489327

[62] Khan J Wang Q Ren Y Eliav R Korczeniewska OA Benoliel R . Exercise induced hypoalgesia profile in rats is associated with IL-10 and IL-1 β levels and pain severity following nerve injury. Cytokine. (2021) 143:155540. doi: 10.1016/j.cyto.2021.155540, PMID: 33902989

[63] Singh SK Krukowski K Laumet GO Weis D Alexander JF Heijnen CJ . CD8+ T cell-derived IL-13 increases macrophage IL-10 to resolve neuropathic pain. JCI Insight. (2022) 7:e154194. doi: 10.1172/jci.insight.154194, PMID: 35260535 PMC8983134

[64] Jeong JY Chung YC Jin BK . Interleukin-4 and interleukin-13 exacerbate neurotoxicity of prothrombin kringle-2 in cortex *in vivo* via oxidative stress. Int J Mol Sci. (2019) 20:1927. doi: 10.3390/ijms20081927, PMID: 31010119 PMC6515094

[65] Ydens E Cauwels A Asselbergh B Goethals S Peeraer L Lornet G . Acute injury in the peripheral nervous system triggers an alternative macrophage response. J Neuroinflamm. (2012) 9:1–17. doi: 10.1186/1742-2094-9-176, PMID: 22818207 PMC3419084

[66] McCormick SM Heller NM . Commentary: IL-4 and IL-13 receptors and signaling. Cytokine. (2015) 75:38–50. doi: 10.1016/j.cyto.2015.05.023, PMID: 26187331 PMC4546937

[67] Li X Xu H Li C Guan Y Liu Y Zhang T . Biological characteristics of tissue engineered-nerve grafts enhancing peripheral nerve regeneration. Stem Cell Res Ther. (2024) 15:215. doi: 10.1186/s13287-024-03827-9, PMID: 39020413 PMC11256578

[68] Kiguchi N Sakaguchi H Kadowaki Y Saika F Fukazawa Y Matsuzaki S . Peripheral administration of interleukin-13 reverses inflammatory macrophage and tactile allodynia in mice with partial sciatic nerve ligation. J Pharmacol Sci. (2017) 133:53–6. doi: 10.1016/j.jphs.2016.11.005, PMID: 28057412

[69] Allen JE . IL-4 and IL-13: regulators and effectors of wound repair. Annu Rev Immunol. (2023) 41:229–54. doi: 10.1146/annurev-immunol-101921-041206, PMID: 36737597

[70] Kuyrukluyıldız U Küpeli İ Bedir Z Özmen Ö Onk D Süleyman B . The effect of anakinra on paclitaxel-induced peripheral neuropathic pain in rats. Turkish J anaesthesiology reanimation. (2016) 44:287., PMID: 28058139 10.5152/TJAR.2016.02212 PMC5207416

[71] Starobova H Monteleone M Adolphe C Batoon L Sandrock CJ Tay B . Vincristine-induced peripheral neuropathy is driven by canonical NLRP3 activation and IL-1β release. J Exp Med. (2021) 218(5):e20201452. doi: 10.1084/jem.20201452, PMID: 33656514 PMC7933984

[72] Kanter AG Ülger H Bozkurt AS Tarakçıoğlu M Özercan İ.H Ulusal H . Investigation into effects of tocilizumab and epoetin beta in rats with experimental sciatic nerve injury model. Tissue Cell. (2024) 88:102357. doi: 10.1016/j.tice.2024.102357, PMID: 38493757

[73] Zhou YQ Liu Z Liu ZH Chen SP Li M Shahveranov A . Interleukin-6: an emerging regulator of pathological pain. J Neuroinflamm. (2016) 13:1–9. doi: 10.1186/s12974-016-0607-6, PMID: 27267059 PMC4897919

[74] Liang G Han Y He H Lu C Zhu C . Case report and brief literature review: possible association of secukinumab with Guillain–Barré syndrome in psoriasis. Front Immunol. (2024) 15:1412470. doi: 10.3389/fimmu.2024.1412470, PMID: 39007153 PMC11239418

[75] Patel SB Roy DR Sweers BWB Coffin MK . Dupilumab, a novel treatment for peripheral neuropathy: A case series. Dermatol Ther. (2025) 15(6):1569–77. doi: 10.1007/s13555-025-01415-0, PMID: 40274710 PMC12092312

[76] Milligan ED Penzkover KR Soderquist RG Mahoney MJ . Spinal interleukin-10 therapy to treat peripheral neuropathic pain. Neuromodulation. (2012) 15:520–6. doi: 10.1111/j.1525-1403.2012.00462.x, PMID: 22672183 PMC3443506

[77] Fang Y Shi B Liu X Luo J Rao Z Liu R . Xiaoyao pills attenuate inflammation and nerve injury induced by lipopolysaccharide in hippocampal neurons in *vitro*. Neural Plasticity. (2020) 2020:8841332. doi: 10.1155/2020/8841332, PMID: 33014035 PMC7525321

[78] Shiue SJ Rau RH Shiue HS Hung YW Li ZX Yang KD . Mesenchymal stem cell exosomes as a cell-free therapy for nerve injury–induced pain in rats. Pain. (2019) 160:210–23. doi: 10.1097/j.pain.0000000000001395, PMID: 30188455

[79] Yang L Cao J Du Y Zhang X Hong W Peng B . Initial IL-10 production dominates the therapy of mesenchymal stem cell scaffold in spinal cord injury. Theranostics. (2024) 14:879. doi: 10.7150/thno.87843, PMID: 38169599 PMC10758068

[80] Skundric DS Lisak RP Rouhi M Kieseier BC Jung S Hartung HP . Schwann cell-specific regulation of IL-1 and IL-1Ra during EAN: possible relevance for immune regulation at paranodal regions. J Neuroimmunology. (2001) 116:74–82. doi: 10.1016/S0165-5728(01)00281-8, PMID: 11311332

[81] Hardy PB Wang BY Chan KM Webber CA Senger JLB . The use of electrical stimulation to enhance recovery following peripheral nerve injury. Muscle Nerve. (2024) 70:1151–62. doi: 10.1002/mus.28262, PMID: 39347555

[82] Hoyng SA De Winter F Tannemaat MR Blits B Malessy MJ Verhaagen J . Gene therapy and peripheral nerve repair: a perspective. Front Mol Neurosci. (2015) 8:32. doi: 10.3389/fnmol.2015.00032, PMID: 26236188 PMC4502351

